# Integrated analysis of the relation to tumor immune microenvironment and predicted value of *Stonin1* gene for immune checkpoint blockage and targeted treatment in kidney renal clear cell carcinoma

**DOI:** 10.1186/s12885-023-10616-9

**Published:** 2023-02-09

**Authors:** Axiu Zheng, Jianrong Bai, Yanping Ha, Yaping Yu, Yonghao Fan, Meihua Liang, Yanda Lu, Zhihua Shen, Botao Luo, Wei Jie

**Affiliations:** 1 Department of Pathology, School of Basic Medicine Sciences; Pathology Diagnosis and Research Center of Affiliated Hospital, Guangdong Medical University, Zhanjiang, 524023 PR China; 2 Department of Pathology, Shanghai Dongfang Hospital, Shanghai, 200120 PR China; 3 Department of Oncology of the First Affliated Hospital; Oncology Institute, Hainan Medical University, Haikou, 571199 PR China

**Keywords:** Stonin1, Kidney renal clear cell carcinoma, Tumor immune microenvironment, Immune checkpoint blockage, Immunotherapy response, Targeted therapeutics

## Abstract

**Background:**

Stonin1 (STON1) is an endocytic protein but its role in cancer remains unclear. Here, we investigated the immune role of *STON1* in kidney renal clear cell carcinoma (KIRC).

**Methods:**

We undertook bioinformatics analyses of the expression and clinical significance of *STON1* in KIRC through a series of public databases, and the role of *STON1* in the tumor microenvironment and the predictive value for immunotherapy and targeted treatment in KIRC were identified with R packages. *STON1* expression was validated in clinical KIRC tissues as well as in KIRC and normal renal tubular epithelial cells.

**Results:**

Through public databases, *STON1* mRNA was found to be significantly downregulated in KIRC compared with normal controls, and decreased *STON1* was related to grade, TNM stage, distant metastasis and status of KIRC patients. Compared with normal controls, STON1 was found to be downregulated in KIRC tissues and cell lines. Furthermore, OncoLnc, Kaplan–Meier, and GEPIA2 analyses also suggested that KIRC patients with high *STON1* expression had better overall survival. The high *STON1* group with enriched immune cells had a more favorable prognosis than the low *STON1* group with decreased immune cells. Single sample Gene Set Enrichment Analysis and Gene Set Variation Analysis indicated that *STON1* creates an immune non-inflamed phenotype in KIRC. Moreover, *STON1* was positively associated with mismatch repair proteins and negatively correlated with tumor mutational burden. Furthermore, Single sample Gene Set Enrichment Analysis algorithm and Pearson analysis found that the low *STON1* group was more sensitive to immune checkpoint blockage whereas the high *STON1* group was relatively suitable for targeted treatment.

**Conclusions:**

Decreased *STON1* expression in KIRC leads to clinical progression and poor survival. Mechanically, low *STON1* expression is associated with an aberrant tumor immune microenvironment. Low *STON1* is likely to be a favorable indicator for immunotherapy response but adverse indicator for targeted therapeutics in KIRC.

**Supplementary Information:**

The online version contains supplementary material available at 10.1186/s12885-023-10616-9.

## Introduction

Renal cell carcinoma (RCC) is a type of cancer generally derived from renal tubular epithelial cells, with 73,820 new cases and 14,770 deaths reported in 2019 [1]. Most cases occur in developed countries, and the morbidity rate in men is two to three times that in women [2]. There are various subtypes of RCC according to the 2016 World Health Organization (WHO) classification of tumors of the urinary system, such as kidney renal clear cell carcinoma (KIRC), chromophobe RCC, papillary RCC, and collecting duct and renal medullary carcinoma [3]. KIRC accounts for 70% of cases of RCC [4].

The current treatment strategy for RCC includes partial nephrectomy, radical nephrectomy, ablative therapies, and targeted therapeutics [5], and its pathogenesis has been widely explored; however, 25% of RCC patients still undergo disease progression or metastasis [6]. Recently, immune checkpoint blockage (ICB) therapy was developed. Monoclonal antibodies against immune checkpoint blockade molecules—such as CTLA-4 (cytotoxic T lymphocyte antigen 4), PD-1 (programmed cell death 1), and PD-L1 (programmed cell death ligand 1)—target the tumor microenvironment (TME) with an obvious objective response rate for KIRC [7]. Nevertheless, the absence of economical and effective indicators for predicting response to ICB treatment remains a hindrance. Thus, there is an urgent need to identify novel biomarkers to guide prognosis, ICB treatment prediction, and clinical management.

*Stonins* are an evolutionally conserved family that mediate the recovery and circulation of vesicles at neuromuscular junctions, and consist of *Stonin1* (*STON1*, also known as germ-line-specific transcription factor) and *Stonin2* (*STON2*) [8]. The human STON1 protein consists of 735 amino acids and has a predicted molecular mass of 83 kDa [9]. Due to the alternative splicing, another two STON1 isoforms with molecular mass of 127 kDa and 129 kDa were displayed in uniport database. Additionally, the level of *STON1* gene methylation is a prognostic marker for the progression and personalized treatment of obesity [10]. Focal adhesion (FA) plays a crucial role in tumor cell motility which are orchestrated by signals cells receive from outside via cell surface receptors. STON1, as a regulator of FA dynamics and cell motility, can facilitates the internalization of the oncogenic proteoglycan neuron-glial antigen 2 (NG2), an FA-associated transmembrane protein serving as a promoter of cellular motility and tumor growth [11], but the potential functions of STON1 in mediating specific molecular mechanisms in carcinomas remain entirely unexplored.

In our current study, the expression of *STON1* in KIRC was explored using online databases combined with clinical tissue array and cell lines. The prognostic value of *STON1* in KIRC was explored using bioinformatics analysis. Additionally, the relationship between *STON1* expression and the tumor microenvironment was primarily presented with Kaplan–Meier survival analysis. Our multidimensional analysis ultimately demonstrated that high *STON1* expression contributed to a non-inflamed KIRC phenotype, and that cases with low expression of *STON1* seem to be sensitive to ICB, whereas high *STON1* cases are more suitable for targeted treatment. Our current research has revealed the potential new role of *STON1* in cancer, special for KIRC.

## Methods

### Expression of *STON1* in KIRC

The Tumor Immune Estimation Resource (TIMER) database (TIMER2.0, http://timer.comp-genomics.org/) is a comprehensive web server for systematical analysis of immune infiltrates across diverse cancer types. We used Diff Exp module to explore *STON1* mRNA expression between pan-cancer and normal tissue, including KIRC. The statistical significance computed by the Wilcoxon test [12]. The independent KIRC cohorts were downloaded from the Gene Expression Omnibus (GEO) database, including GSE16441 (platform: GPL6480), GSE16449 (platform: GPL6480), and GSE71963 (platform: GPL6480), with the “GEO2R” online tool [13] (https://www.ncbi.nlm.nih.gov/geo/). STON1 protein expression was assessed using the UALCAN database (http://ualcan.path.uab.edu/index.html) which provides protein expression analysis option using data from Clinical Proteomic Tumor Analysis Consortium (CPTAC) [14].

### Tissue microarray and immunohistochemical staining (IHC)

The tissue microarray data (Cat: HKidE180Su02) was obtained from Shanghai Outdo Biotech Co., LTD (Shanghai, China), which enrolled 30 normal kidney tissues and 150 KIRC tissues. The clinicopathological parameters of the KIRC patients in the tissue microarray are presented in Supplementary Table 1. The IHC was performed using a DAKO automatic immunohistochemistry instrument with the programs of the “Autostainer Link 48 Usage Guide”. The array was incubated with primary antibodies against STON1 (Cat: ABP53586, Abbkine, Wuhan, China) at 1:200 dilution overnight at 4 °C. Non-immune IgG was used as a negative control. Antigenic sites were visualized using a UltraSensitive SP kit (Cat: KIT-9709, Maxin, Fuzhou, China) and DAB kit (Cat: GK600511, GeneTech, Shanghai, China). The STON1 scores were calculated as follows: 0, negative; 1, weak; 2, moderate; and 3, strong. The percentage of positive cells was scored as follows: 1, 0–25% positive cells; 2, 26–50% positive cells; 3, 51–75% positive cells, and 4, 76–100% positive cells. The total immunoreactive scores were determined by the nuclear staining score plus the cytoplasm membrane staining score.

### Analysis of the relationships between *STON1* and clinical phenotype and prognosis in KIRC

RNA sequencing data and the related clinicopathological data were downloaded from TCGA using UCSC Xena (https://xena.ucsc.edu/), an online explorer that allows users to explore functional genomic data sets for correlations between genomic and phenotypic variables. A total of 491 patients obtained from UCSC Xena were enrolled in the cohort after exclusion criteria were applied. The exclusion criteria were as follows: 1) patients without follow-up records; 2) patients without a diagnosis of KIRC; 3) the primary tumor could not be evaluated; and 4) the stage and grade were not reported. Finally, 491 patients were divided into high and low STON1 expression groups according to the median expression value of *STON1*. The OncoLnc database (http://www.oncolnc.org/) is a platform for survival analysis with TCGA data [15]. Kaplan–Meier Plotter (https://kmplot.com/analysis/) is an accessible online website with the purpose of identifying survival biomarkers. The prognosis value of each marker based on different immune cells backgrounds was also assessed [16]. We applied OncoLnc and Kaplan–Meier to identify the independent prognostic value of *STON1* in KIRC. We also analyzed the prognostic value of *STON1* expression in KIRC patients using the Gene Expression Profiling Interactive Analysis (GEPIA) browser (http://gepia2.cancer-pku.cn/#survival) [17].

### The immunological role of *STON1* in the KIRC tumor microenvironment (TME)

We first identified the infiltration score of 35 immune cells by exploring Assistant for Clinical Bioinformatics (https://www.aclbi.com), which consists of fifteen modules, as well as the immune score, stroma score, and microenvironment score. After selecting the Immunity module, we divided the TCGA-KIRC cohort into low and high *STON1* groups according to the median value of *STON1*, excluding cases where the metastasis and grade (Mx and Gx) could not be evaluated. The statistical difference of two groups was compared through the Wilcoxon test and the consequence was presented with box plot by using Xcell algorithm. The cBio Cancer Genomics Portal (http://cbioportal.org) is an open-access resource for interactive exploration of multidimensional cancer genomics data sets [18]. Kidney was selected as the primary site in the query module of the database, which contained 17 data sets with different sample quantity. Then, KIRC dataset containing 538 tumor samples (Kidney Renal Clear Cell Carcinoma-TCGA, Firehose Legacy) was screened for further analysis, and input the gene symbol *STON1*. Finally, the co-expression module was used to analyze the correlation between *STON1* and expression of immune cell biomarker. The anti-tumor immune response is a multistep coordinated process called the cancer-immunity cycle, which determines the final direction of immune activation or suppression [19]. The cancer-immunity cycle, including seven steps based on specific marker gene sets (Supplemental Table 2), was obtained from the TIP (Tracking Tumor Immunophenotype) database (http://biocc.hrbmu.edu.cn/TIP/) [20]. The enrichment score, indicating anti-cancer immunity, was calculated with the Single sample Gene Set Enrichment Analysis (ssGSEA) algorithm using the R package “GSEABase” and was presented with a boxplot by employing the online tool SangerBox 3.0 (http://vip.sangerbox.com/home.html). The correlation heatmap between *STON1* expression and the enrichment score of the cancer-immunity cycle pathways was presented with the R package “ggcorrplot”. In addition, we selected four stromal pathways with immunosuppressive effects from the previous literature, including epithelial–mesenchymal transition (EMT) markers and the pan-fibroblast TGF-β response signature (Pan-FTBRS) [21]. The Gene Set Variation Analysis (GSVA) algorithm was performed to calculate the enrichment score of these signatures.

### Correlation of tumor mutational burden (TMB), microsatellite instability, copy number variation (CNV), tumor mRNA stemness index (mRNAsi) and *STON1*

Spearman correlation analysis was conducted to identify the correlation between *STON1* expression and microsatellite instability and a marginal scatter plot was acquired by the “ggpubr” R package for the KIRC mRNA profile. The relationship between the expression of *STON1*, the tumor mutational burden (TMB) score and the tumor mRNA stemness index (mRNAsi) was displayed by the Assistant for Clinical Bioinformatics with TCGA module and CSCs module, an open integrated database described above (https://www.aclbi.com/). The TIMER database with SCNA module was employed to display the correlation between copy number variants of *STON1* and immune cells using a two-sided Wilcoxon rank-sum test [22].

### Analysis of the value of *STON1* in predicting immunotherapy and targeted therapy response in KIRC

The enrichment score of 19 ICB-response-associated gene signatures downloaded from the previous literature were calculated with the ssGSEA algorithm [21]. These signatures contain 18 positive pathways and one negative pathway (Supplemental Table 3). We further verified the correlation between *STON1* and the expression of each immune checkpoint biomarkers with Gene_Corr module in the TIMER2.0 database. The different IC50 values between the low and the high *STON1* group were calculated using the Assistant for Clinical Bioinformatics (https://www.aclbi.com/) with the IC50 module.

### Validation *STON1* expression in normal renal tubular epithelial cell line and KIRC cell lines

Normal renal tubular epithelial cell line HK-2, KIRC cell line A498, ACHN and 786-O were presented by the Department of Urology, Affiliated Hospital of Guangdong Medical University. All cell lines were maintained in Dulbecco's modified eagle medium (ThermoFisher Scientific China, Shanghai) supplemented with 10% fetal bovine serum (ThermoFisher Scientific China), and cells were cultured at 37 °C, 5% CO_2_ in saturation humidity. qRT-PCR and western blotting were used to detect the expression of *STON1*. For qRT-PCR, the following primers pairs were used, *STON1* (NM_001198595.2) upstream, 5’-GCCCAAATATTTCCTGCAGAGTC-3’, *STON1* downstream, 5’-CTGAGGCCAGGAAGGTTCAG-3’; *GAPDH* (NM_002046.7) upstream, 5’-TCGGAGTCAACGGATTTGGT-3’, *GAPDH* downstream, 5’-TTCCCG TTCTCAGCCTTGAC-3’. For western blotting, the primary antibody against STON1 (Cat: Abp53586, Abbkine) and GAPDH (Cat: 2118, CST China, Shanghai) were used.

### Statistical analysis

All mRNA expression data from public databases was normalized by log_2_ transition. A columnar scatter plot was created by GraphPad Prism (Version 8, GraphPad Software, CA, USA). The immunoreactive scores of STON1 in tissue arrays were calculated using the Wilcoxon signed-rank test. Unpaired t-test was adopted to compare *STON1* mRNA levels in cell lines using GraphPad Prism (Version 8). The chi-squared test was performed using IBM SPSS Statistics (version 25) to analyze the association between the *STON1* mRNA level and clinicopathological parameters. The correlation analysis between *STON1*, the mismatch repair system, and the TMB was performed using Pearson’s test in RStudio software (version 4.0.3). *P* < 0.05 was considered statistically significant.

## Results

### *STON1* expression was significantly reduced in KIRC

**Fig. 1:**
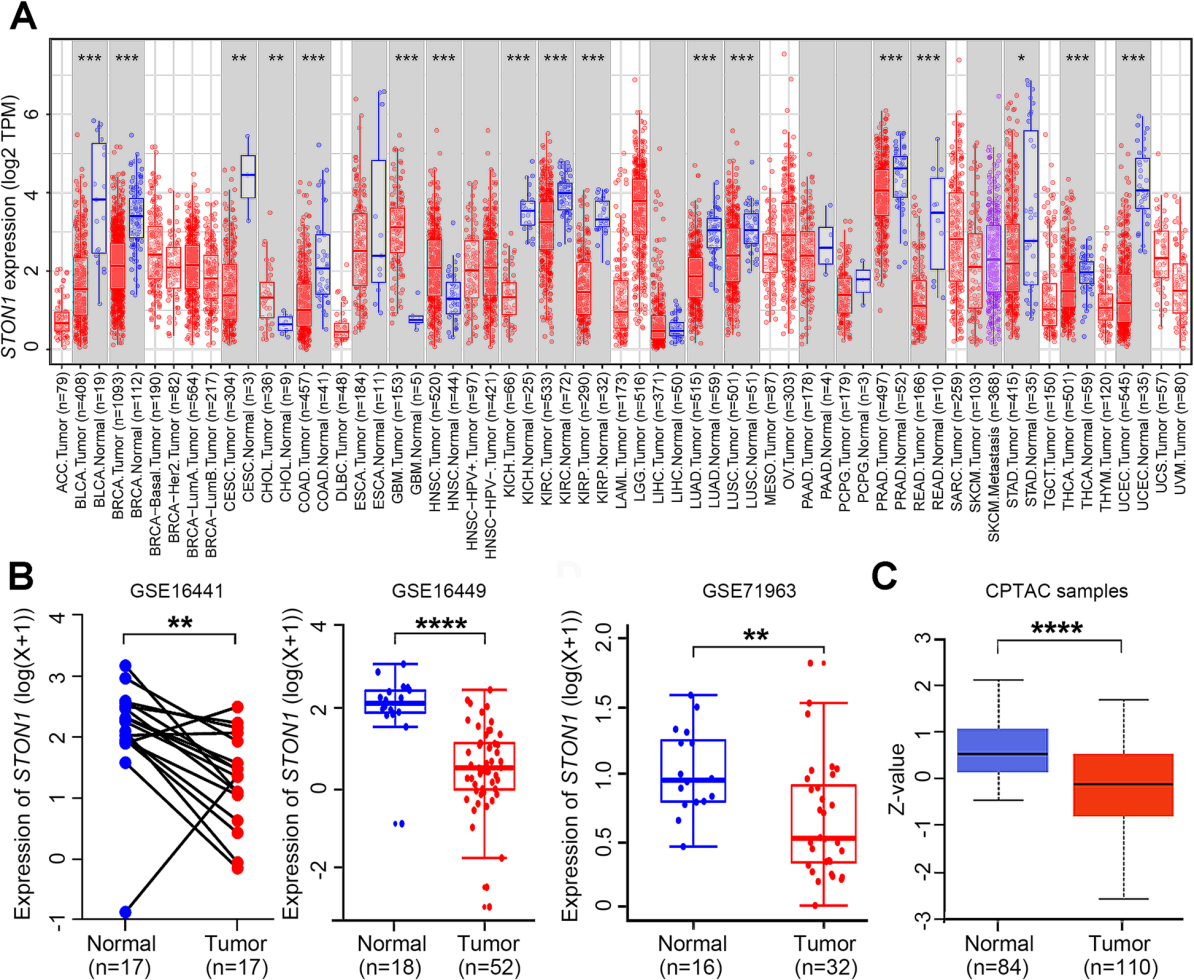
*STON1* expression is downregulated in KIRC. **A** *STON1* mRNA expression in tumor and normal tissues of different human cancer types in TCGA database (the Wilcoxon test). **B** *STON1* mRNA level in KIRC tumors and adjacent normal tissue in GSE16441, GSE16449, and GSE71963 from GEO database. **C** Protein expression of STON1 in CPTAC samples. *P < 0.05, **P < 0.01, ***P < 0.001, ****P < 0.0001

**Fig. 2:**
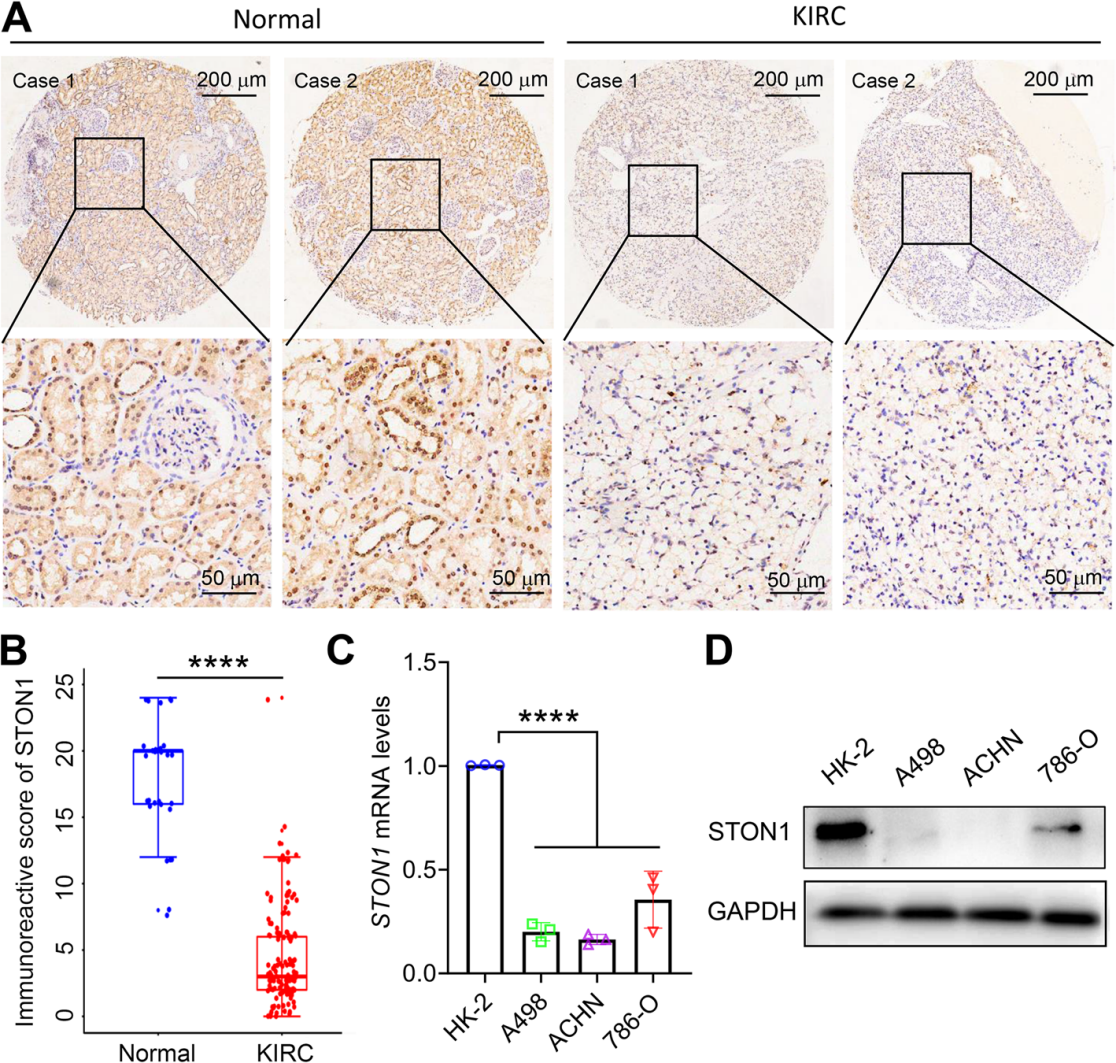
Expression of *STON1* in KIRC tissue microarray and cell lines. **A** Representative images of STON1 protein in clinical KIRC and control tissues. **B** The immunoreactive score of STON1 presented by boxplot with t-test. ****P < 0.0001. **C** Expression of *STON1* mRNA in normal renal tubular epithelial cells and KIRC cell lines. qRT-PCR was used to detect the expression of *STON1* mRNA in normal renal tubular epithelial cell HK-2 and KIRC cell line A498, ACHN and 786-O. ****P < 0.0001. **D** Representative western blotting bands for STON1 protein in normal renal tubular epithelial cell HK-2 and KIRC cell line A498, ACHN and 786-O. For the original bands, please see Additional file 4

We first identified the transcription level of *STON1* in different TCGA tumors with the TIMER2.0 database. A total of 33 human cancer types were explored. We found that most cancers including KIRC, kidney renal papillary cell carcinoma (KIRP), kidney chromophobe (KICH), breast invasive carcinoma (BRCA), colon adenocarcinoma (COAD), lung adenocarcinoma (LUAD), lung squamous cell carcinoma (LUSC), prostate adenocarcinoma (PRAD), stomach adenocarcinoma (STAD), thyroid carcinoma (THCA), and uterine corpus endometrial carcinoma (UCEC)—showed a decreased *STON1* mRNA level, while head and neck squamous cell carcinoma (HNSC) displayed an upregulated *STON1* mRNA level (Fig. 1a). Moreover, results from three GEO datasets, GSE16441, GSE16449, and GSE71963, also revealed aberrantly downregulated *STON1* mRNA in KIRC (*P* < 0.01, Fig. 1b). Finally, the protein level of STON1 in KIRC was also found to be reduced using the CPTAC database (*P* < 0.0001) (Fig. 1c). We further validated the protein expression with our tissue microarray, and the results showed that KIRC samples displayed significantly lower immunoreactive scores compared with paired adjacent normal tissue (*P* < 0.0001, Fig. 2a, b). According to the results of in vitro cytological experiments, *STON1* was again found to be downregulated in KIRC cell lines compared with normal renal tubular epithelial cell HK-2 (Fig. 2c, d, Supplementary Fig. 1).

### Correlation between *STON1* expression and clinicopathological characteristics of KIRC patients

**Fig. 3:**
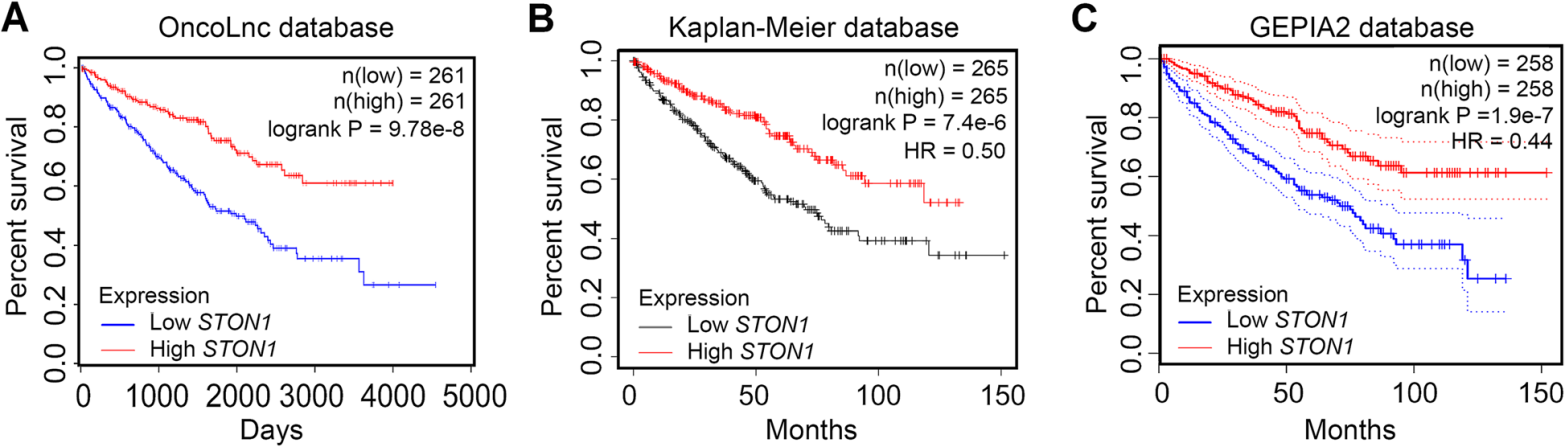
High expression of *STON1* indicated a better outcome in KIRC patients. Kaplan–Meier curves of overall survival according to high and low expression of *STON1* in TCGA data in KIRC patients by exploring the OncoLnc database (**A**), Kaplan–Meier database (**B**), and GEPIA2 database (**C**)

The KIRC GDC Expression Matrix was downloaded from the UCSC Xena, which is one of the most comprehensive clinical oncology databases [23]. A total of 491 KIRC patients were included in the cohort after excluding non-KIRC diagnoses and those with missing clinical information. They were classified into either a high expression group (*n* = 246) or a low expression group (*n* = 245) according to the median value of *STON1*. Notably, the *STON1* mRNA levels were related to grade (*P* = 0.022), TNM stage (*P* = 0.039), distant metastasis (*P* = 0.019), and vital status (*P* = 5.46 × 10^−6^). However, no correlation was found between *STON1* mRNA levels and age (*P* = 0.136), gender (*P* = 0.949), invasion depth (*P* = 0.074), and lymph node metastasis (*P* = 0.385) (Table 1). To further analyze the prognostic value of *STON1*, we performed a survival analysis using three oncological databases. The results indicated that *STON1* expression levels were strongly associated with overall survival (OS) in KIRC. Patients with high expression of *STON1* had a better outcome compared with those with low levels of *STON1* in the OncoLnc database (logrank *P* = 9.78 × 10^−8^) (Fig. 3a). Similar results were acquired from the Kaplan–Meier database, and GEPIA2 with TCGA data (Fig. 3b–c).

**Table 1:** Association between *STON1* expression and clinicopathological parameters of KIRC patients

**Classification**	***n***	***STON1* expression**		**χ2**	***P* value**
		**Low**	**High**		
**Age**					
< 60	228	106	122	2.22	0.136
** ≥ **60	263	140	123		
**Gender**					
Female	165	83	82	0.004	0.949
Male	326	163	163		
**Grade**					
1	10	4	6	9.641	0.022^*^
2	214	92	122		
3	195	106	89		
4	72	44	28		
**Stage**					
I	239	106	133	8.362	0.039^*^
II	52	30	22		
III	120	61	59		
IV	80	49	31		
**Invasion Depth**					
T1	245	110	135	6.945	0.074
T2	63	36	27		
T3	172	92	80		
T4	11	8	3		
**Lympth nodal metastasis**					
Nx	245	117	128	1.911	0.385
N0	232	120	112		
N1	14	9	5		
**Distant metastasis**					
M0	414	198	216	5.469	0.019^*^
M1	77	48	29		
**Status**					
Alive	325	139	186	20.674	5.44E-06^****^
Dead	166	107	59		

^*^P < 0.05, ****P < 0.0001 was considered statistically significant

### The prognostic value of *STON1* based on the levels of infiltration of various immune cells

**Fig. 4:**
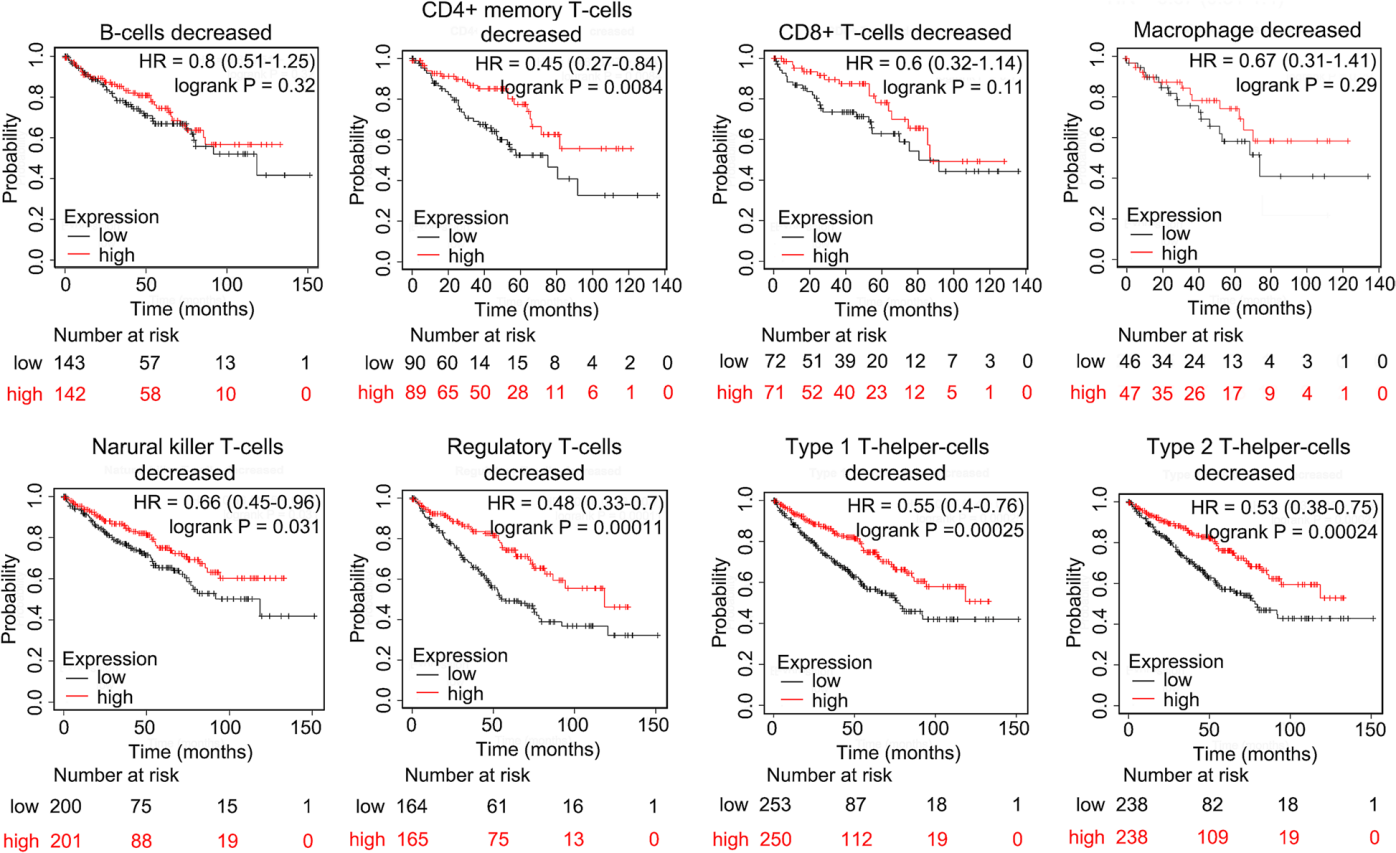
The prognostic value of *STON1* in cases with decreased numbers of eight immune cells

**Fig. 5:**
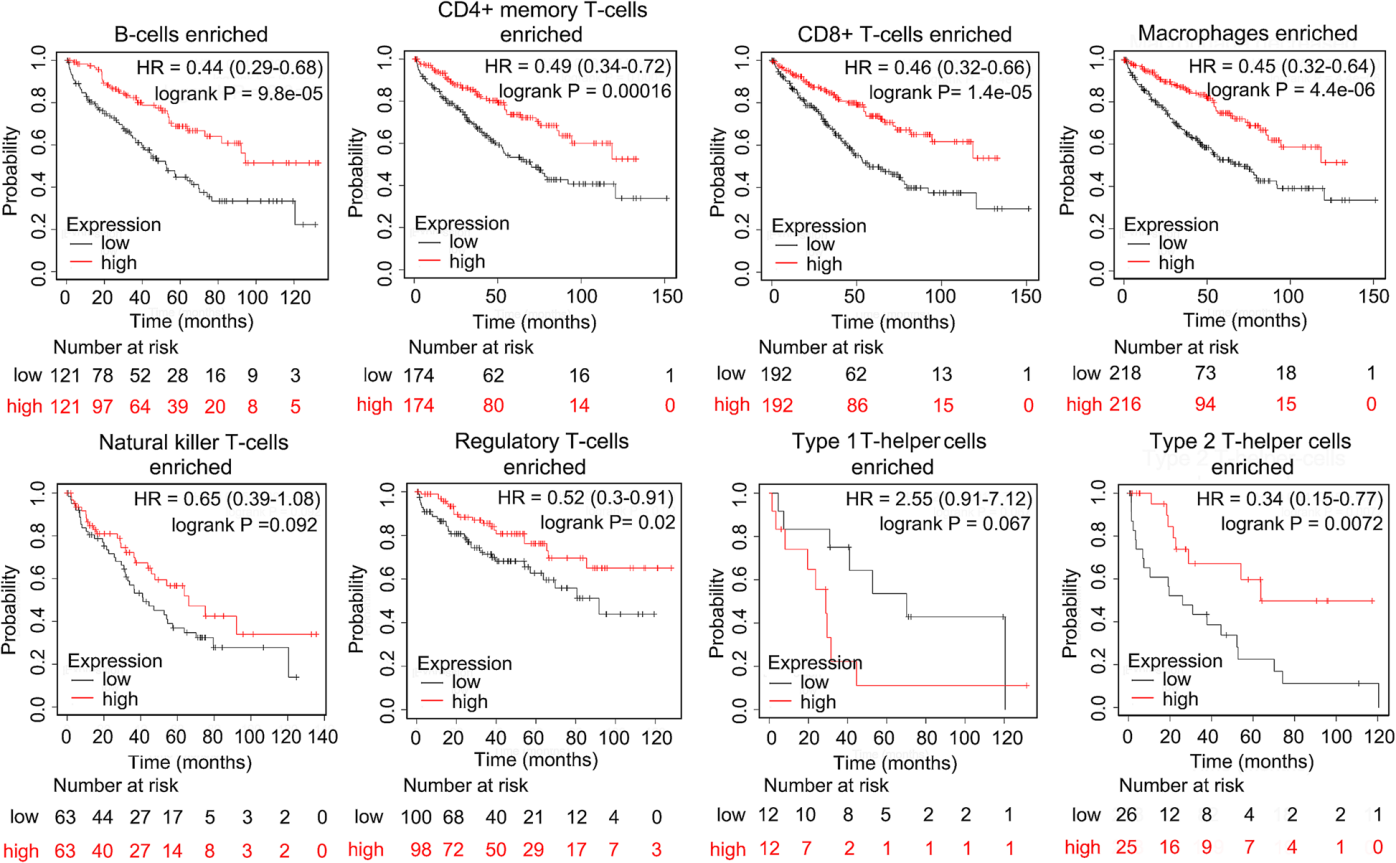
The prognostic value of *STON1* in cases enriched with eight immune cells

Kaplan–Meier survival curves of the differential expression of *STON1* between KIRC cohorts either enriched with immune cells or with decreased immune cells were explored. The high *STON1* groups of KIRC cohorts with decreased CD4 + memory T cells (HR = 0.45, *P* = 0.0084), natural killer T cells (HR = 0.66, *P* = 0.031), regulatory T cells (Treg cell, HR = 0.48, *P* = 0.00011), type 1 T-helper cells (HR = 0.55, *P* = 0.00025), and type 2 T-helper cells (HR = 0.53, *P* = 0.00024), indicating favorable prognosis (Fig. 4). Similarly, high *STON1* levels in the KIRC cohorts enriched with B cells (HR = 0.44, *P* = 9.8 × 10^−5^), CD4 + memory T cells (HR = 0.49, *P* = 0.00016), CD8 + T cells (HR = 0.46, *P* = 1.4 × 10^−5^), and macrophages (HR = 0.45, *P* = 4.4 × 10^−6^) exhibited a better OS, except natural killer T cells and type 1 T-helper cells (Fig. 5). Interestingly, high *STON1* expression in KIRC with enriched type 1 T-helper cells displayed a poor OS, although it was not statistically significant (HR = 2.55, *P* = 0.067) (Fig. 5).

### Low* STON1* expression forms an immune inflamed phenotype in KIRC

**Fig. 6:**
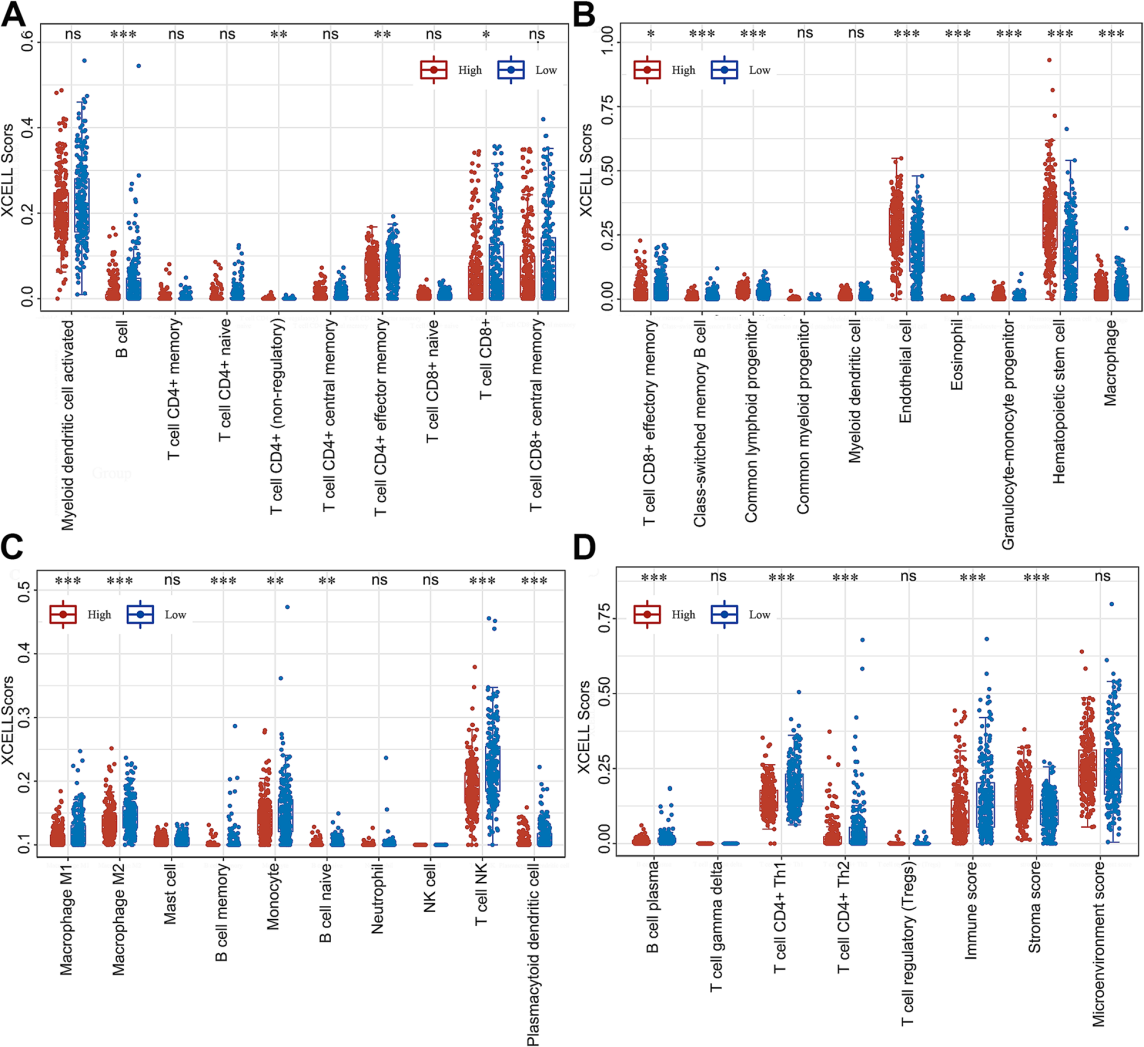
The correlation between *STON1* and the tumor microenvironment. **A–D** The percentage abundance of tumor-infiltrating immune cells in each sample, with different colors and types of immune cells. (The ordinate represents the percentage of immune cell content in a single sample; high, high expression of *STON1* group; low, low expression of *STON1* group; the Wilcoxon test). *P < 0.05, **P < 0.01, ***P < 0.001; ns, not statistically significant

**Fig. 7:**
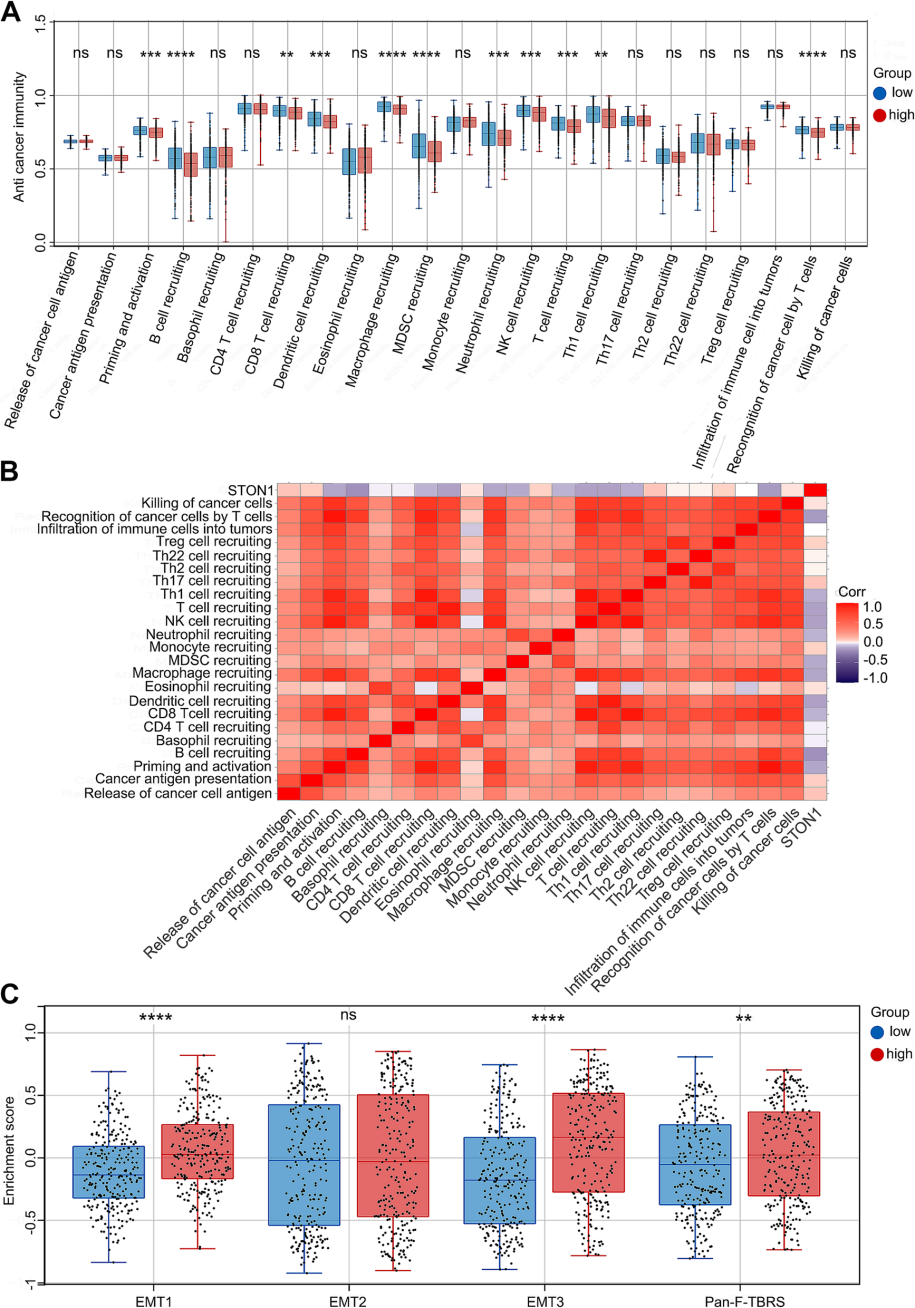
The different immune phenotypes between the low and high *STON1* groups. **A** Enrichment score of cancer-immunity cycle steps between the two groups. (High, high expression of the *STON1* group; low, low expression of the *STON1* group; ssGSEA analysis and the Wilcoxon test; the abscissa represents different anti-tumor immune cycle steps and the ordinate represents the enrichment score based on special gene signatures; **P < 0.01; ***P < 0.001; ****P < 0.0001; ns, not statistically significant). **B** The correlation between *STON1* expression and the enrichment score of cancer-immunity cycle. **C** Enrichment score of four stromal pathways between the two groups. (High, high expression of *STON1* group; low, low expression of *STON1* group; GSVA analysis and the Wilcoxon test; **P < 0.01; ****P <0.0001; ns, not statistically significant)

Given that *STON1* play totally different role in certain immune environment, we further analyzed the potential association between *STON1* and immune cells. The majority of the cancer-related immune cell scores for B cells, CD4 + effector/memory T cells, CD8 + T cells, CD8 + effector/memory T cells, class-switched memory B cells, macrophages, M1 macrophages, M2 macrophages, memory B cells, monocytes, native B cells, natural killer (NK) T cells, plasmacytoid dendritic cells, plasma B cells, CD4 + T-helper 1 (Th1) cells and CD4 + T-helper 2 (Th2) cells were lower in the high *STON1*-expressing KIRC group (Fig. 6a–d). Additionally, we noted the immune score was higher in the low *STON1* group, whereas the stroma score was relatively downregulated (Fig. 6d). To further confirm the relationship between *STON1* expression and immune cell infiltration levels in KIRC, we used cBioportal database to explore the correlations between *STON1* expression and various immune infiltration associated markers [24]. Our results showed there was a significant correlation between *STON1* expression and the most of biomarker sets of CD8 + T cell, T cell (general), B cell, TAM, M1 macrophage, M2 macrophage, Th1, Th2, Tfh, Th17, Treg, T cell exhaustion (Supplemental Table 4). Cancer-Immunity Cycle is a multistep fine-regulated network, so we explored the function of *STON1* in anti-tumor immune activities to compare the difference between the low and high *STON1* groups. As shown in Fig. 7a, the low *STON1* group manifested an intensive effect of anti-cancer immunity activities, especially priming and activation, the recruiting of B cells, CD8 T cells, dendritic cells, macrophages, myeloid derived suppressor cells (MDSCs), neutrophils, NK cells, T cells, and Th1 cells, and the recognition of cancer cells by T cells. The heatmap also indicated that *STON1* expression was obviously negatively related to most of these cancer-immunity cycle pathway signatures (Fig. 7b, Supplemental Table 5). These results imply that low *STON1* prompts an immune inflamed phenotype in KIRC. Furthermore, a previous report had proposed that activation of stromal pathways results in immunosuppressive effects of anti-cancer immunity [21]. Consistent with the above results, the enrichment scores of the EMT1 pathway, the EMT3 pathway, and the pan-F-TBRS pathway were significantly higher in the high *STON1* group (Fig. 7c).

### Correlations of *STON1* levels with TMB, mismatch repair genes, CNV, and tumor stem index in KIRC

**Fig. 8:**
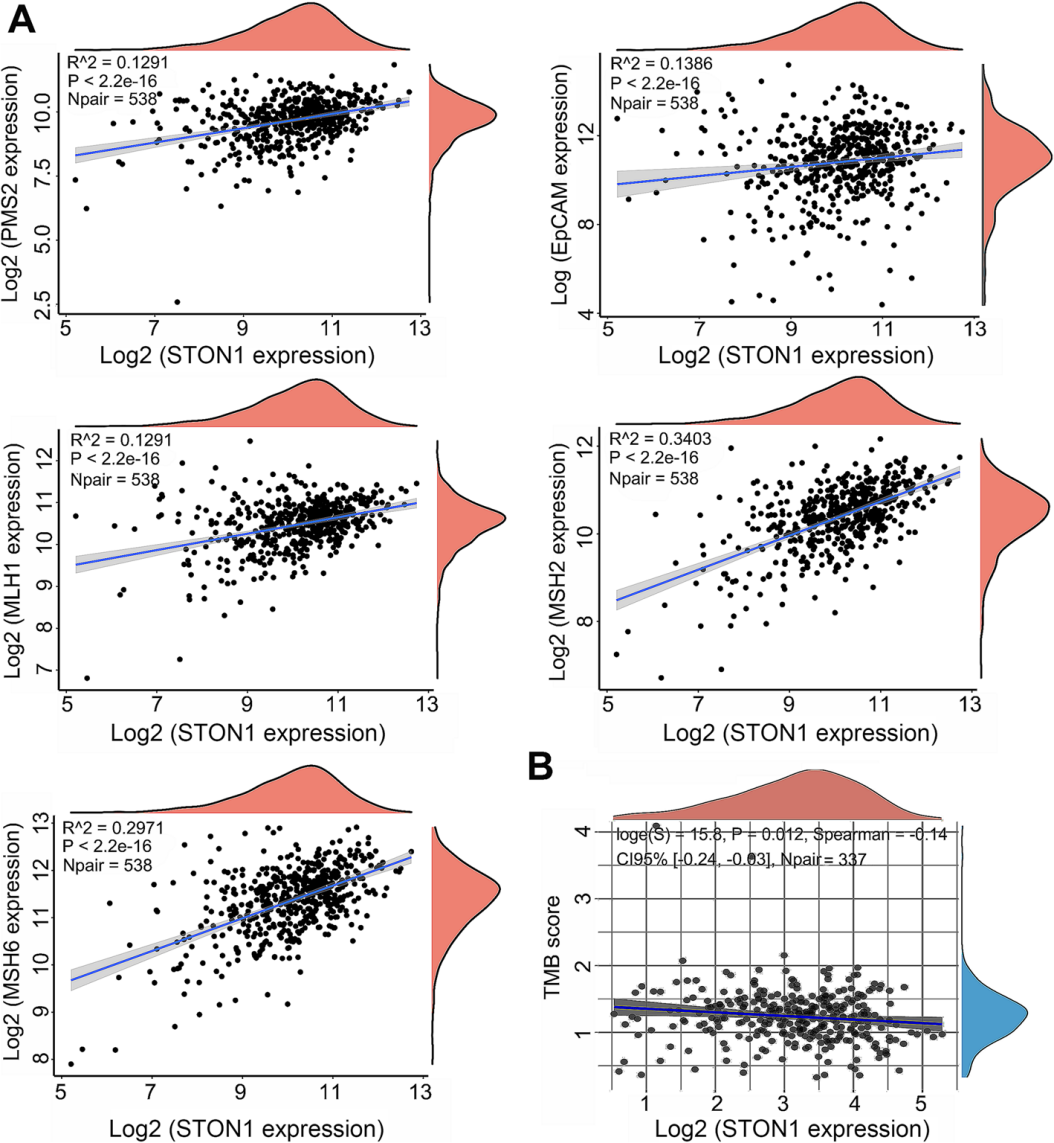
Correlation between *STON1* and the mismatch system and tumor mutational burden (TMB). **A** The *STON1* mRNA level was positively associated with MLH1, PMS2, MSH2, MSH6, and EpCAM, as presented in marginal scatter plots. **B** Relationship between the *STON1* mRNA level and TMB in marginal scatter plots. The expression values of all genes are presented as logarithmic values

**Fig. 9:**
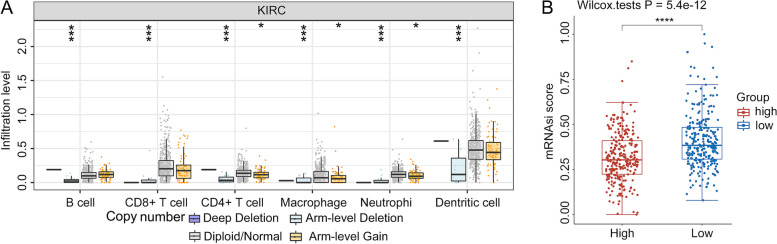
Association between copy number variants (CNV) of *STON1*, immune cells, and cancer stem index. **A** The infiltration level of six immune cell subtypes related to *STON1* CNV. **B** The stem characteristics of tumor cells between the low *STON1* group and the high *STON1* group. *P < 0.05, ***P < 0.001, ****P < 0.0001

Given the sensitive connection between TMB, microsatellite instability (MSI), and immunotherapeutic response, we focused on their relationship with *STON1* expression levels. Marginal scatter plots revealed that alterations in *STON1* expression were always accompanied by changes in mismatch repair genes (MLH1, PMS2, MSH2, MSH6, and EpCAM). Moreover, the four mismatch genes seemed to be positively associated with *STON1* (Fig. 8a, *P* < 2.2 × 10^−16^). The *STON1* mRNA level was weakly negatively associated with the TMB score (*P* = 0.012) (Fig. 8b). In addition, the infiltration level of six immune cells was distinctly downregulated by arm-level deletion of *STON1* (Fig. 9a). Cancer stem cells (CSCs) are characterized by the ability to generate all cell types in specific cancer samples. Compared with non-tumor stem cells, CSCs have self-protection mechanisms, such as DNA damage repair and inhibition of apoptotic pathways, that in turn lead to tumor progression, metastasis, drug resistance and increased self-renewal [25]. We found that the low *STON1* group displayed a higher mRNAsi score (Fig. 9b), a marker associated with malignant biological processes in CSCs and more tumor dedifferentiation [26].

### Decreased *STON1* predicted a better response to ICB

**Fig. 10:**
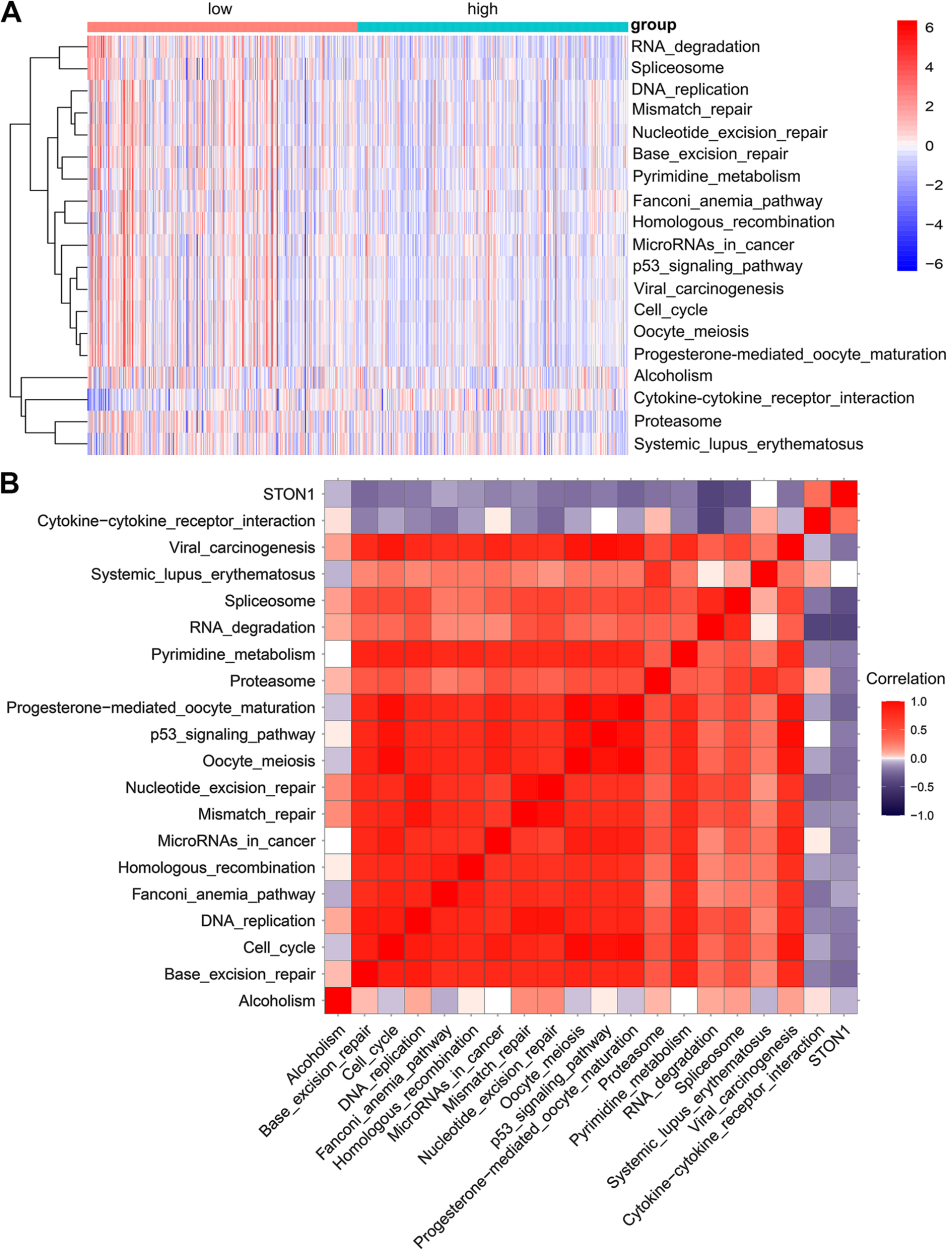
Sensitivity difference to ICB response between two groups. **A** The heatmap of the enrichment score of 19 special signatures for ICB response between the two groups. (High, high expression of *STON1* group; low, low expression of *STON1* group; ssGSEA analysis and the Wilcoxon test; the bar plot on the right represents the enrichment score based on special gene signatures). **B** The correlation between *STON1* expression and the enrichment score of 19 special signatures for ICB response

**Fig. 11:**
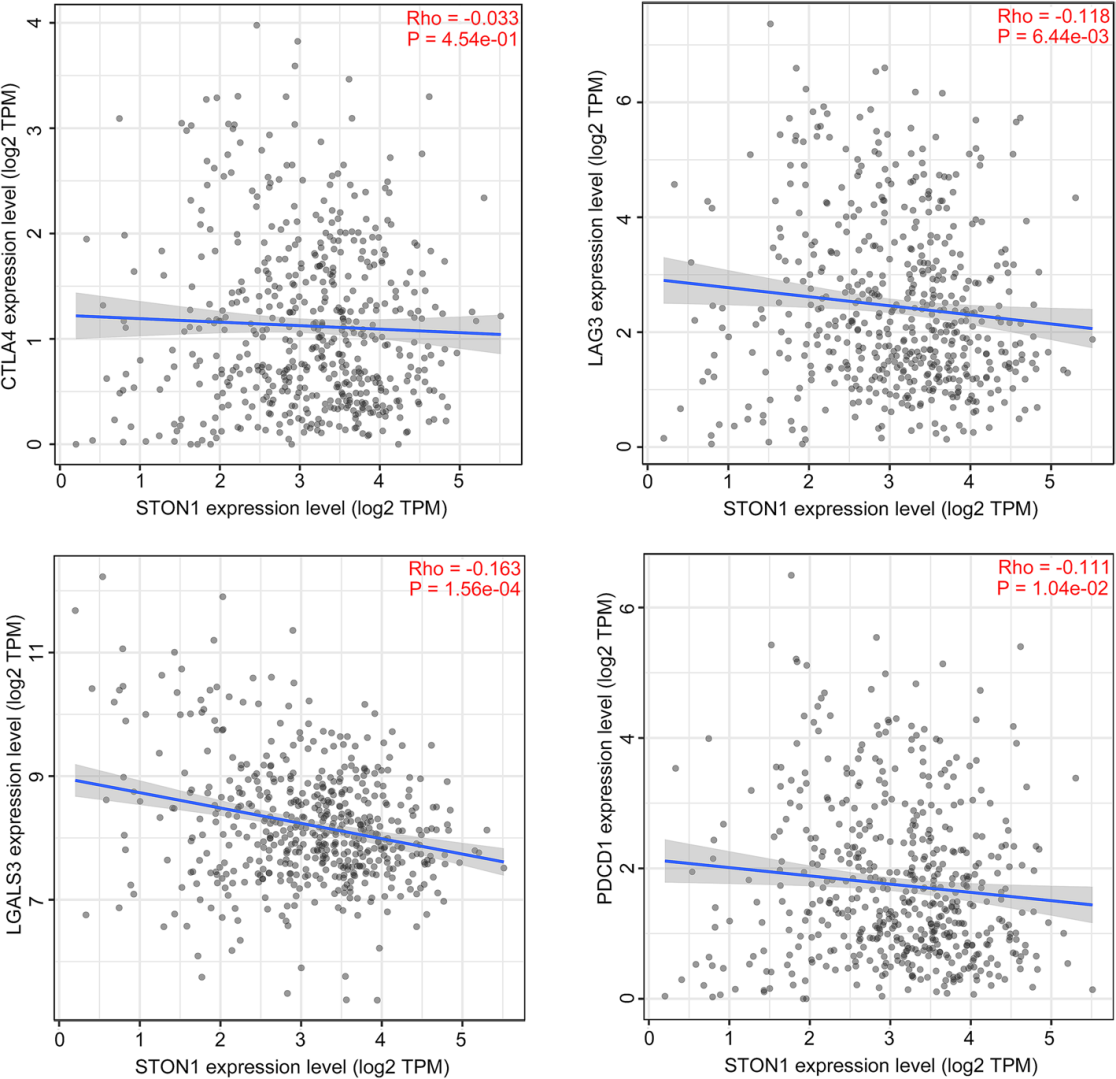
The relationship between *STON1* expression and four immune checkpoints

The immune inflamed tumor phenotype is essential for KIRC patient response to ICB [27, 28]. We further analyzed the different ICB response between the low and high *STON1* groups based on the efficacy of ICB-response-related signatures. The enrichment score for pathways related to a positive response to ICB was higher in the low *STON1* group, such as RNA degradation, spliceosome, DNA replication, mismatch repair, and nucleotide excision repair (Fig. 10a). In contrast, the enrichment score for cytokine–cytokine receptor interaction, giving a negative response to ICB, was upregulated in the high *STON1* group (Fig. 10a). Moreover, correlation analysis showed that *STON1* expression was negatively correlated with the enrichment score of most of the eighteen ICB-response-related pathways (Fig. 10b, Supplemental Table 6). Given that immune checkpoints play an indispensable role in immunotherapy [29], our study found that *STON1* expression was negatively correlated with the expression of immune checkpoints such as LAG3 (Lymphocyte activating 3), LGALS3 (Galectin 3), PDCD1 (Programmed cell death 1), and CTLA4 (Cytotoxic T-lymphocyte associated protein 4) (Fig. 11).

### The high *STON1* group was more sensitive to targeted treatment of KIRC

**Fig. 12:**
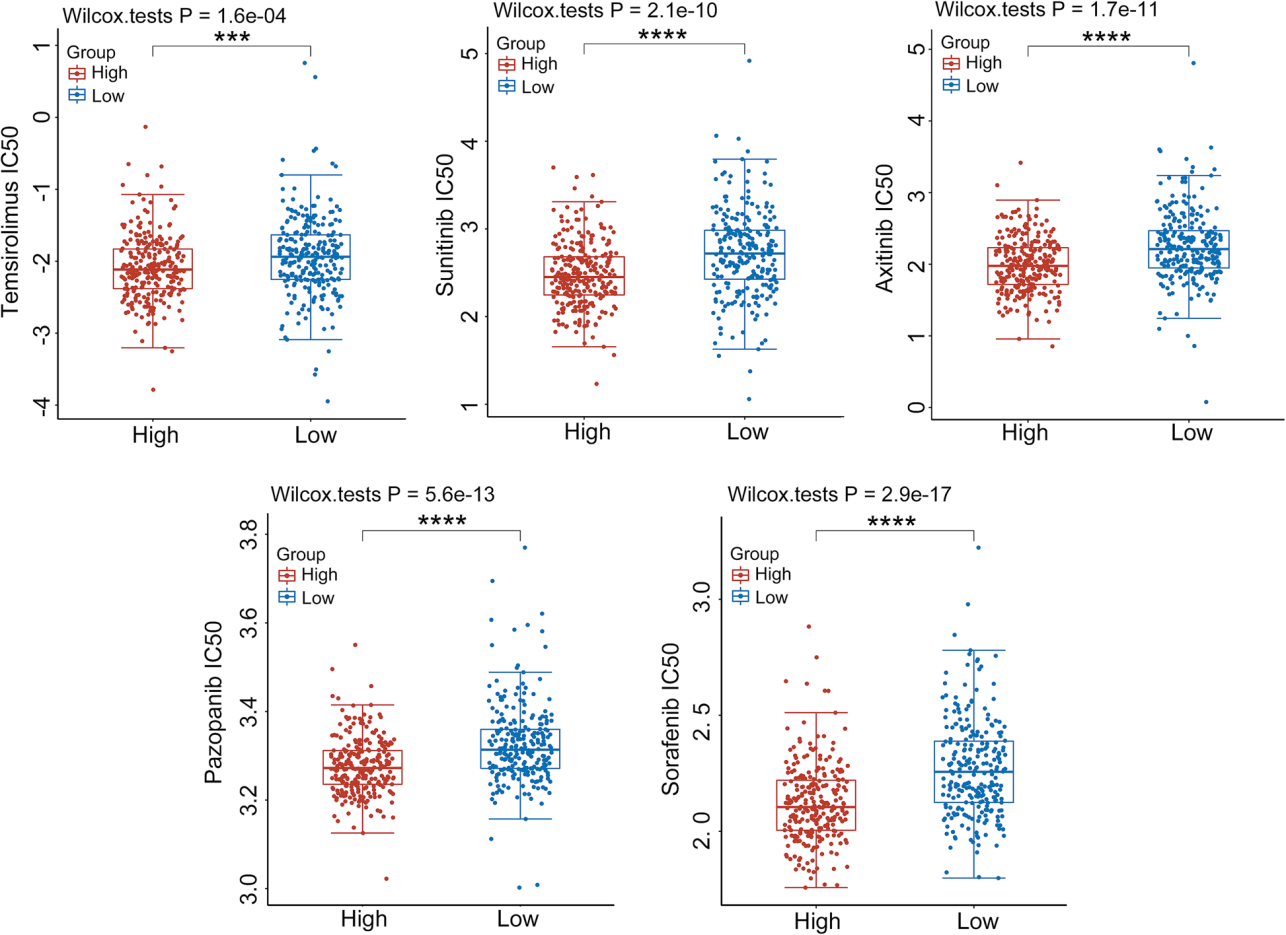
The sensitivity for five targeted agents among the two groups in KIRC. High, high expression of *STON1* group; low, low expression of *STON1* group; the vertical axis represents the distribution of IC50 scores; ***P < 0.001, ****P < 0.0001

Currently, molecular targeting drugs for metastatic KIRC mainly include inhibitors for vascular endothelial growth factor (VEGF) signaling (Sunitinib, Pazopanib, Axitinib, Sorafenib) and the mammalian target of rapamycin (mTOR) inhibitor (Temsirolimus) [30–32]. We therefore employed the disparate sensitivity of these agents between the low and high *STON1* groups. We found that the half maximal inhibitory concentration (IC50) score of the low *STON1* group was higher than that of the high *STON1* group in KIRC patients treat with temsirolimus (high_mean_ *vs* low_mean_ = -2.08 *vs* -2.01, *P* < 0.001), which indicated that the high *STON1* group was more sensitive to this drug, as well as sunitinib (high_mean_ *vs* low_mean_ = 2.48 *vs* 2.59, *P* < 0.0001), axitinib (high_mean_ *vs* low_mean_ = 1.98 *vs* 2.12, *P* < 0.001), pazopamib (high_mean_ *vs* low_mean_ = 3.28 *vs* 3.30, *P* < 0.001), sorafenib (high_mean_ *vs* low_mean_ = 2.12 *vs* 2.19, *P* < 0.001) (Fig. 12, Supplemental Table 7).

## Discussion

Although early-stage KIRC patients can be cured by partial or complete nephrectomy with a better outcome, approximately 25% of KIRC patients still suffer from recurrence or metastasis [1, 5]. Recently, immune checkpoint blockers for cancer therapy, such as combination immunotherapy of nivolumab plus ipilimumab, have shown promise in KIRC patients with rapid progression in clinical individualized treatment [33], but some patients displayed a low or no immune response [34]. Therefore, further identification of novel biomarkers to predict the clinical therapy response and survival of KIRC patients remains challenging. In our current study, *STON1,* a protein-coding gene involved in vesicle transport, was identified as a target gene at the rs13405728 locus in polycystic ovary syndrome [35]. A previous study reported that alterations in focal adhesion dynamics, cellular motility, and signaling were induced by the absence of *STON1* [11]. The above processes are closely correlated with tumor migration. However, to date, no reports have published the underlying functions of *STON1* in cancer, especially immune functions. To our knowledge, this is the first report to reveal that *STON1* is decreased in most human cancer types, including KIRC, and is strongly associated with grade, stage, distant metastasis, and vital status in KIRC. IHC analysis and western blotting further indicated the lower expression level of STON1 protein in KIRC tissues and cell lines. Furthermore, *STON1* overexpression favored prognosis in KIRC. The above results indicate that *STON1* may act as a tumor suppressor in KIRC.

Tumor immunotherapy is a prominent milestone of the oncotherapy era. Currently, many indicators have been used to predict the ICB treatment response, such as the number of tumor-infiltrating lymphocytes (TIL), the proportion of CD28 + , CD39 + , and CD96 + TILs, PD-L1 expression, PD-1 expression, the gut microbiome, the TMB, and MSI [19]. However, these indicators are not economical or effective to predict the immunotherapy response because of the complex detection process. With the advent of ICBs, there is an urgent need to determine novel markers to predict immunotherapy responses in clinical practice. In our study, *STON1* was identified as a promising indicator to predict the ICB treatment response and prognosis for KIRC patients.

We preliminarily analyzed the prognostic value of *STON1* enriched or decreased with eight different immune cells. Compared with KIRC cohorts with decreased immune cells, the high *STON1* group enriched with the anti-cancer immune cells B cells, CD4 + T cells, CD8 + T cells, and macrophages demonstrated a favorable prognosis. In line with the above results, the high *STON1* group with decreased levels of Treg or Th2 cells—which are considered immunosuppressors [36, 37]—had a more significant *P* value than the high *STON1* group enriched with Treg cells or Th2 cells. Interestingly, the high *STON1* group enriched with Th1 cells showed a poor OS, although the *P* value was not statistically significant. We speculate that this was because of inadequate samples of KIRC patients. Thus, we were interested in the correlation between *STON1* and TME in KIRC.

CD4 + T cells and CD8 + T cells that contribute to an inflammatory environment act as guardians in anti-tumor immunity [38]. Interferon-γ producing Th1 cells secrete TNF, resulting in tumor destruction. Th1 cells can not only recruit NK cells and macrophages near to the tumor site to exert anti-tumor effects, but also act as enhancers to promote tumor-specific cytotoxic T-lymphocyte (CTL) responses [39]. In contrast, a previous report showed that CD4 + T lymphocytes in a Th2-type tumor microenvironment can promote metastasis by regulating the pro-tumor properties of tumor-associated macrophages (TAMs), as opposed to limiting or eradicating malignant cells by engaging cytotoxic mechanisms [37]. We further analyzed the different infiltration levels of 35 immune cell subtypes between the low *STON1* and high *STON1* groups. Compared with the high group, most infiltration levels of the 35 immune cells were higher in the low *STON1* group, especially for CD8 + T cells, NK T cells, and CD4 + Th1 T cells, as well as the immune score. Furthermore, tumors infiltrated with more activated immune cells (such as CD8 + T and Th1 cells) could have a better response to immunotherapy [40–42]. In the current study, we primarily speculated that the low *STON1* group enriched with immune cells may be a good indicator for ICB treatment. Meanwhile, arm-level deletion of *STON1* also significantly affected the abundance level of immune cells. Additionally, the activities of the cancer-immunity cycle were intense in the low *STON1* group, including priming and activation, recruitment of B cells, CD8 T cells, dendritic cells, and macrophages, and the infiltration of immune cells into tumors. In contrast, the epithelial–mesenchymal translation pathways and the pan-fibroblast TGF-b response signature (Pan-FTBRS) with immunosuppressive effects [21] were significantly activated in the high *STON1* group. In brief, low *STON1* expression shapes an immune inflamed phenotype in KIRC which is essential for immunotherapy.

Mismatch repair genes are related to the human mismatch repair response, which is responsible for the repair of base mismatches that occur during DNA replication [43]. TMB is a novel indicator of mutation quantity [44]. Tumors with mismatch repair possess the capacity to increase the mutational burden, resulting in increased immune checkpoint protein expression, including PD-1 and PD-L1. Furthermore, the immunogenic neoepitopes generated by the imbalance of the mismatch system significantly improved the immunotherapy response rate [45]. Interestingly, the mRNA level of *STON1* was positively associated with mismatch repair (MMR) genes in our current study. As the *STON1* expression decreased, the TMB score increased. Hence, *STON1* may be a candidate prognostic biomarker for evaluating the efficacy of immunotherapy responses and further experiments should be conducted to confirm this theory.

KIRC is one of the most immune-infiltrated cancers of all human solid cancer types [46]. The emergence of immune checkpoint targets provides a new direction for tumor immunotherapy. In recent years, CTLA4 inhibitors, PD-l inhibitors, PD-L1 inhibitors, and PD-L1 inhibitors plus tyrosine kinase inhibitors (TKI) in progressed KIRC patients have been approved by the FDA and achieved great curative effect [47]. We conducted a comprehensive bioinformatics analysis to reveal the detail of *STON1* levels as an indicator for ICB response in KIRC. A total of 19 ICB-response-related signatures were employed to predict different response activities between the two groups. As expected, *STON1* was significantly negatively correlated with 18 pathways that reflect a positive response to ICB and was positively associated with the cytokine–cytokine receptor interaction that is negative for ICB therapy. In addition, the immune checkpoint is necessary for the response to immunotherapy. CTLA4 inhibits T-cell activation to some extent, thus, anti-CTLA4 antibody immunotherapy can enhance the anti-tumor immune effects of T cells [48]. An anti-PD1 (PDCD1) agent series is approved by the FDA and used in various carcinomas with better immune response [49]. Our results indicated that low *STON1* expression correlated with the high expression of these immune checkpoints. Furthermore, we focused on several targeted agents, including temsirolimus, sunitinib, axitinib, pazopamib, sorafenib and found that the IC50 score of these drugs were significantly higher in the high *STON1* group which indicated that targeted therapy could be a treatment option for the high *STON1* group. In summary, ICB application is more suitable for the low *STON1* group, whereas cases with high expression of *STON1* are more sensitive to targeted treatment.

We attempted to validate the expression and prognostic value of *STON1* in our clinical KIRC cohorts and cell lines. Our results also showed that STON1 protein was downregulated in KIRC tissues compared with normal controls. In vivo experiments, we also found that *STON1* was down-regulated in KIRC cell lines compared to normal renal tubular epithelial cells both at mRNA and protein levels. However, because of the incomplete clinical parameters in our tumor cohort, especially the small number of samples in the death group in the follow-up data, we could not obtain satisfactory statistical results. Therefore, a clinical cohort with a larger sample size is required to validate these consequences, and further cytological functional studies need to verify the specific role and molecular mechanism of *STON1* in KIRC in future. Of notable, we detected STON1 protein with the molecular mass between 100 to 130 kDa but not the classical 83 kDa in the KIRC cell lines, the related spicing mechanism need illuminate in future.

## Conclusions

Collectively, lower *STON1* expression in KIRC indicates tumor progression and worse survival outcome. Our current study provides new insights into the function of *STON1* in the tumor microenvironment. High *STON1* expression may shape an immune non-inflamed phenotype. Importantly, *STON1* is relatively suitable as an indicator of ICB and targeted therapy response.

## Supplementary Information

**Additional file 1: Supplemental Table 1.** The clinicopathological data of tissue microarray.

**Additional file 2: Supplemental Table 2.** The gene sets for the cancer-immunity cycle analysis.

**Additional file 3: Supplemental Table 3.** The signatures related to response of immunotherapy.

**Additional file 4: Supplemental Figure 1.** Original bands for western blotting of STON1 and GAPDH in cell lines.

**Additional file 5: Supplemental Table 4.** Correlation analysis between *STON1* and relate genes and markers of immune cells in KIRC by cBioportal database.

**Additional file 6: Supplemental Table 5.** Correlations between *STON1* and anti-cancer immunity in TCGA cohort.

**Additional file 7: Supplemental Table 6.** Correlations between *STON1* and immunotherapy associated signatures in TCGA cohort

**Additional file 8: Supplemental Table 7.** The IC50 score between high and low expression of* STON1* groups in KIRC.

## Data Availability

The datasets used and/or analyzed during the current study are available in the following repositories, GEO database (https://www.ncbi.nlm.nih.gov/geo/), accession numbers: GSE16441, GSE16449 and GSE71963, UCSC Xena (https://xena.ucsc.edu/), TCGA database (https://portal.gdc.cancer.gov/).

## Abbreviations

CNV: Copy number variant
EMT: Epithelial–mesenchymal transition
GEO: Gene Expression Omnibus
GEPIA: Gene Expression Profiling Interactive Analysis
GSVA: Gene Set Variation Analysis
ICB: Immune checkpoint blockage
IHC: Immunohistochemical staining
KIRC: Kidney renal clear cell carcinoma
MMR: Mismatch repair
mRNAsi: mRNA stemness index
NG2: Neuron-glial antigen 2
OS: Overall survival
Pan-FTBRS: Pan-fibroblast TGF-β response signature
RCC: Renal cell carcinoma
ssGSEA: Single sample Gene Set Enrichment Analysis
STON1: Stonin 1
TCGA: The Cancer Genome Atlas
TIMER: The Tumor Immune Estimation Resource
TMB: Tumor mutational burden
TME: Tumor microenvironment
