# Supplementary material for: Integrated analysis of the relation to tumor immune microenvironment and predicted value of Stonin1 gene for immune checkpoint blockage and targeted treatment in kidney renal clear cell carcinoma
Source: BMC Cancer. 2023 Feb 9;23:135. doi: 10.1186/s12885-023-10616-9 (PMC9912524; doi:10.1186/s12885-023-10616-9)
Supplement: Supplementary file 4 — Additional file 4: Supplemental Figure 1. Original bands for western blotting of STON1 and GAPDH in cell lines. [file 12885_2023_10616_MOESM4_ESM.pptx]

## Slide 1
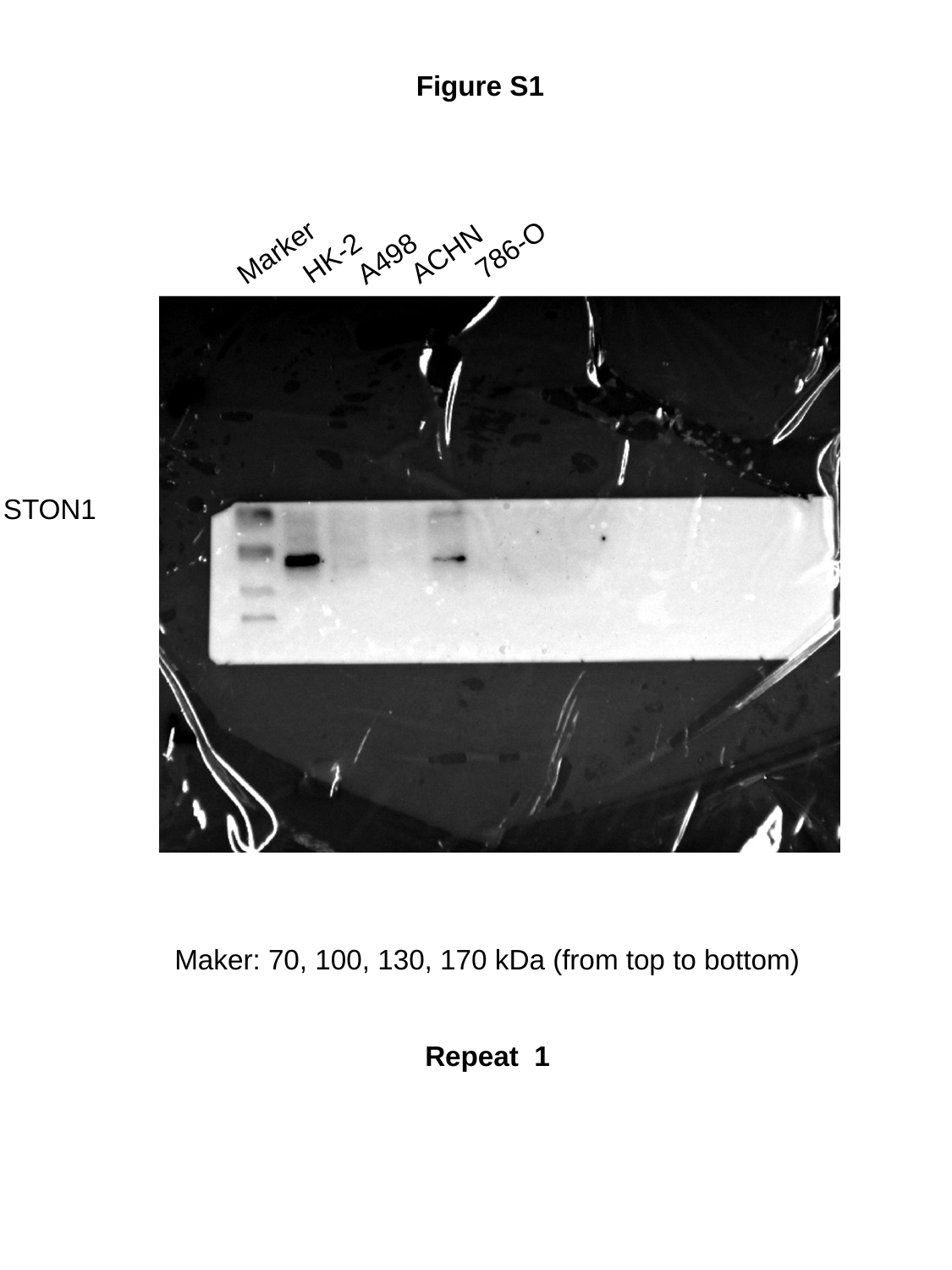

Figure S1
786-O
ACHN
A498
HK-2
Marker
STON1
Maker: 70, 100, 130, 170 kDa (from top to bottom)
Repeat 1

## Slide 2
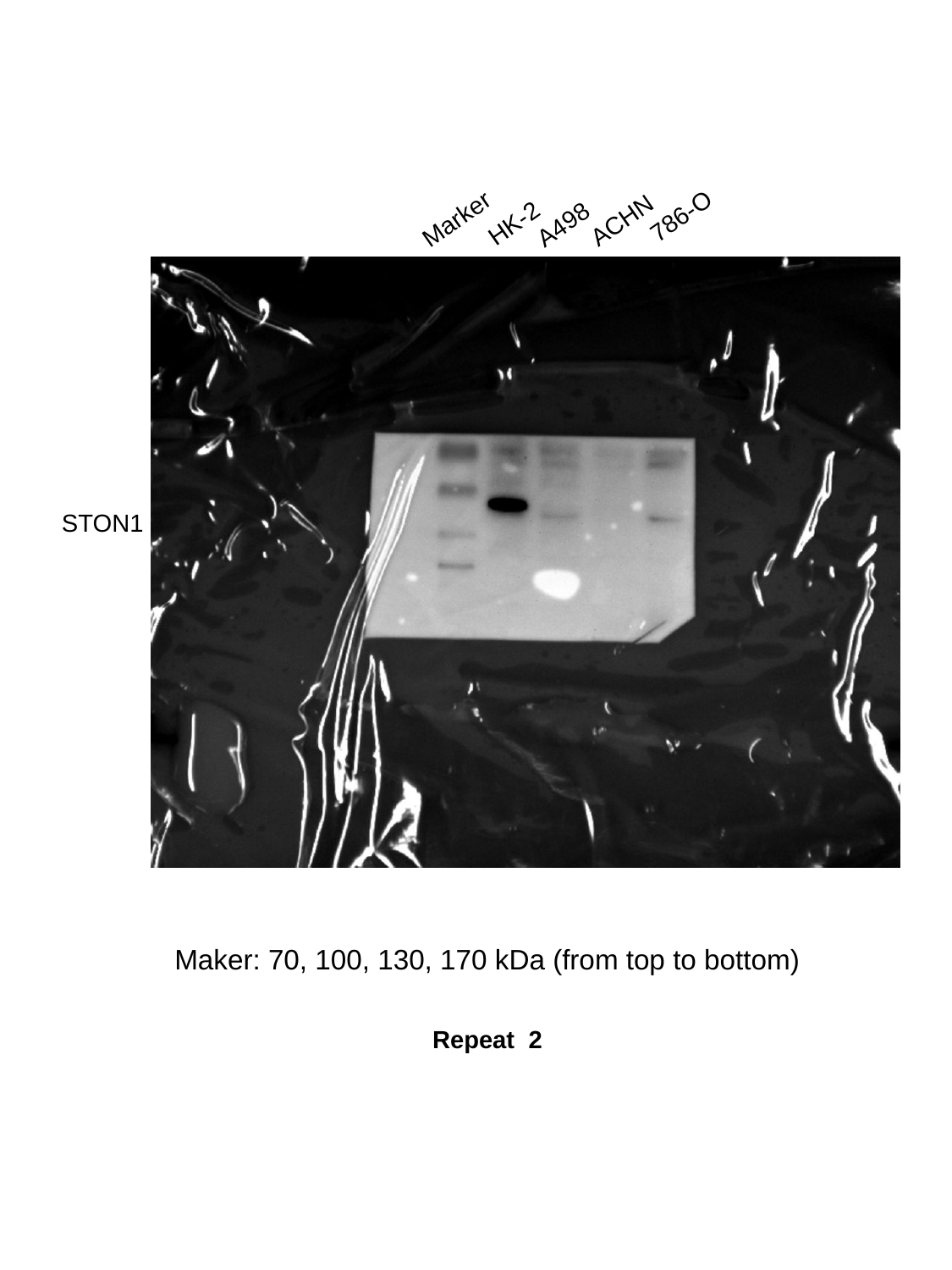

786-O
ACHN
A498
HK-2
Marker
STON1
Maker: 70, 100, 130, 170 kDa (from top to bottom)
Repeat 2

## Slide 3
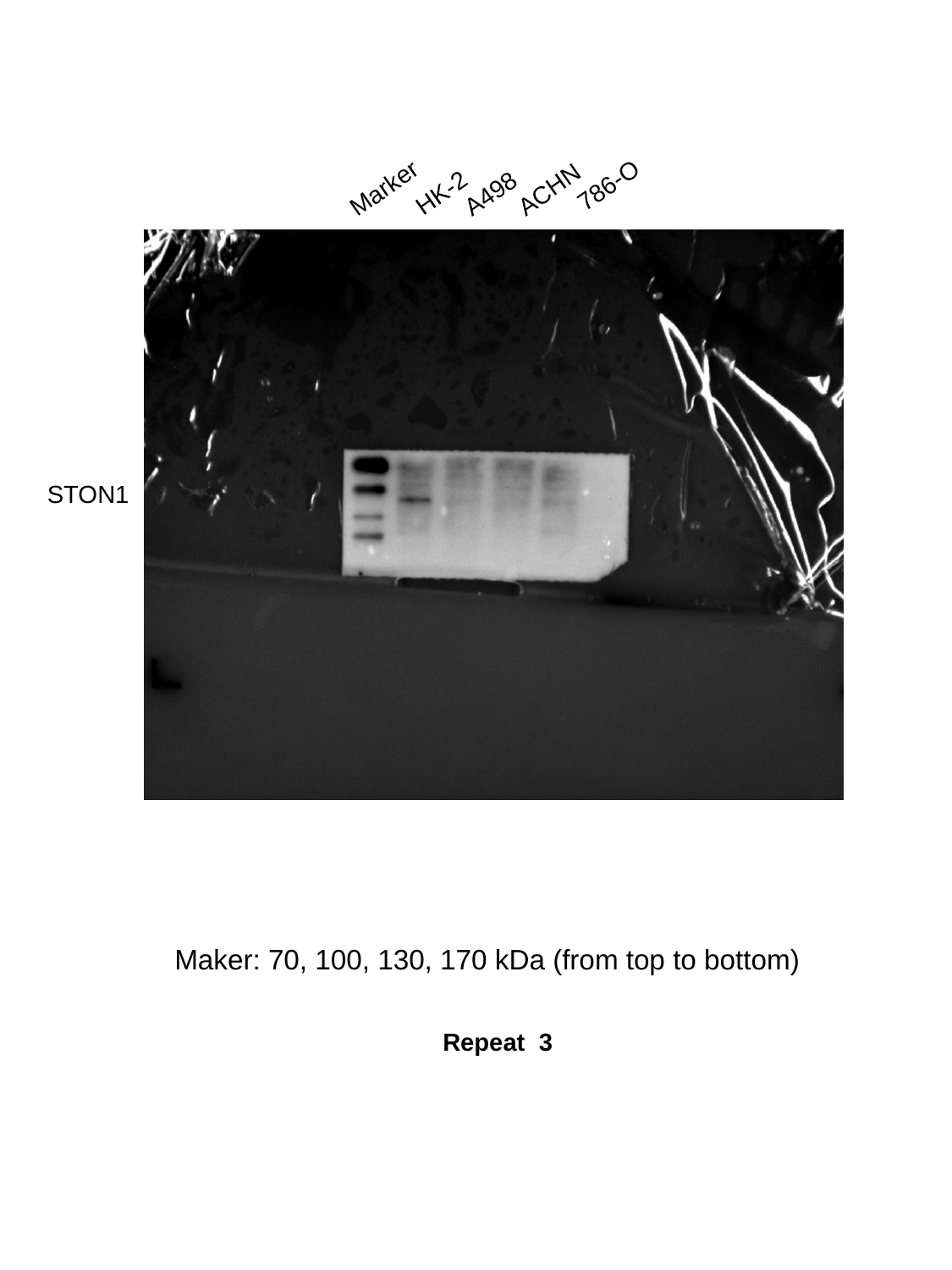

786-O
ACHN
A498
HK-2
Marker
STON1
Maker: 70, 100, 130, 170 kDa (from top to bottom)
Repeat 3

## Slide 4
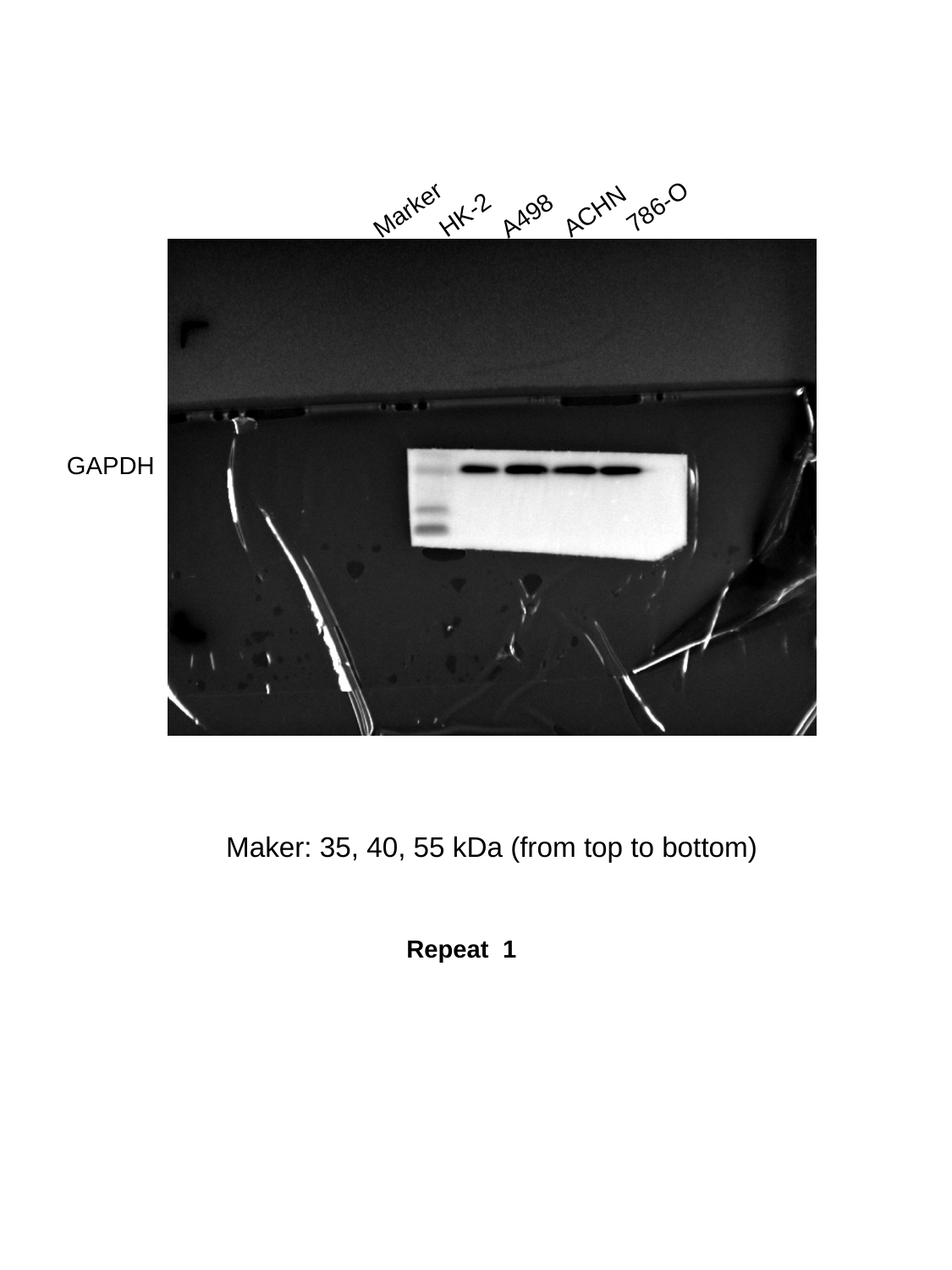

786-O
ACHN
A498
HK-2
Marker
GAPDH
Maker: 35, 40, 55 kDa (from top to bottom)
Repeat 1

## Slide 5
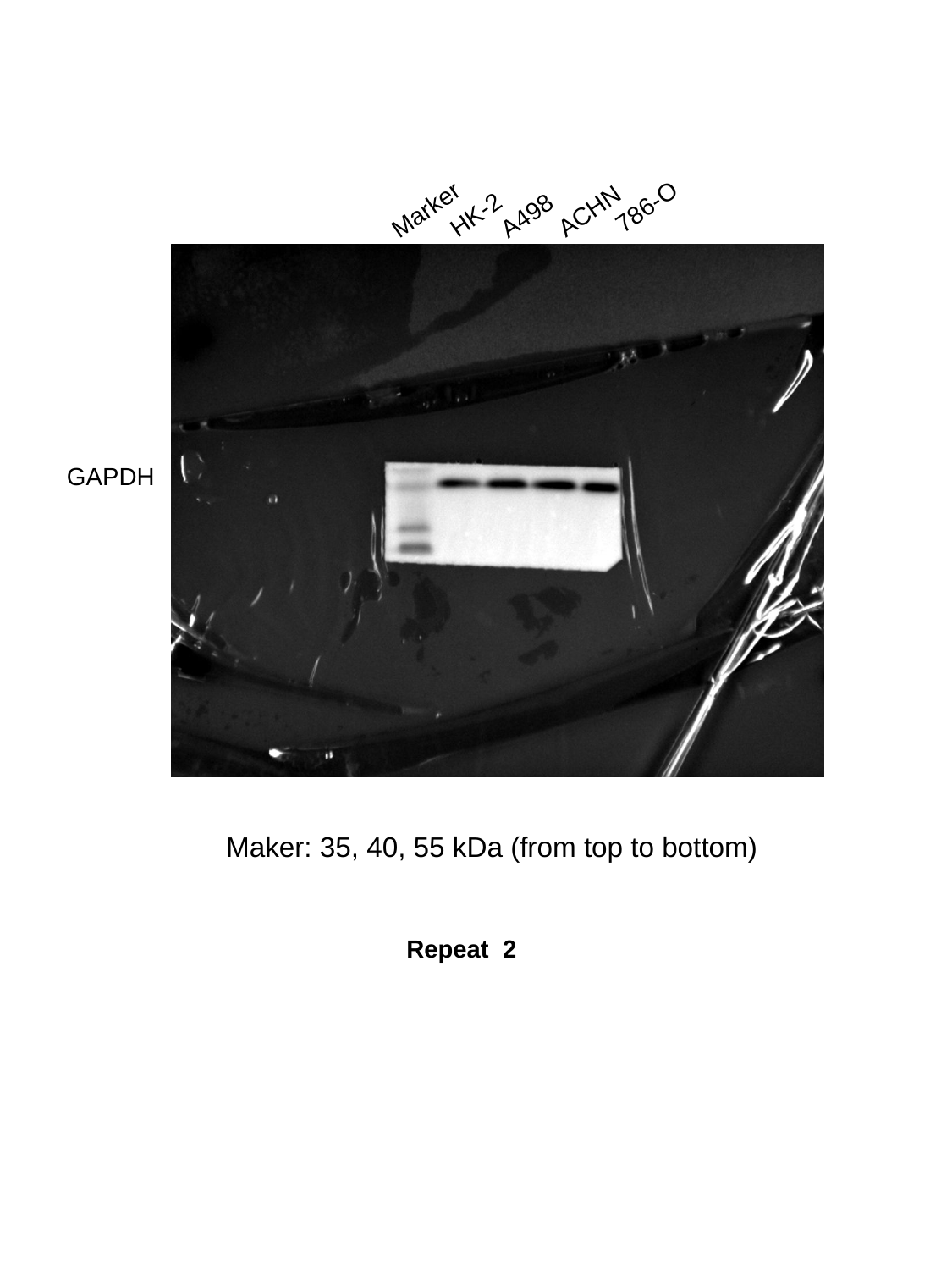

786-O
ACHN
A498
HK-2
Marker
GAPDH
Maker: 35, 40, 55 kDa (from top to bottom)
Repeat 2

## Slide 6
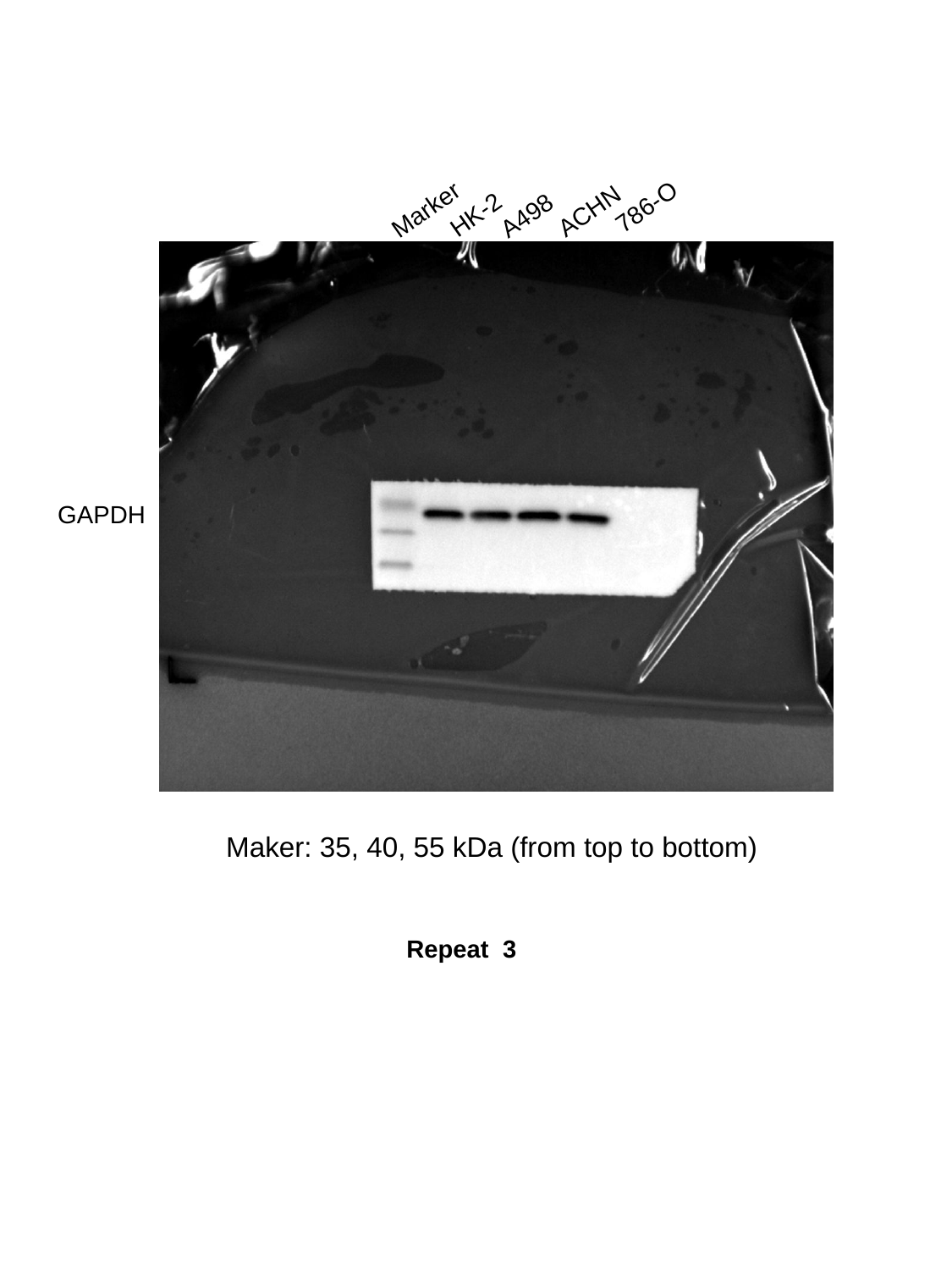

786-O
ACHN
A498
HK-2
Marker
GAPDH
Maker: 35, 40, 55 kDa (from top to bottom)
Repeat 3
